# The pseudoenzyme ADPRHL1 affects cardiac function by regulating the ROCK pathway

**DOI:** 10.1186/s13287-023-03507-0

**Published:** 2023-10-26

**Authors:** Lei Tian, Tianwei Guo, Fujian Wu, Rui Bai, Sinan Ai, Hongyue Wang, Yuanxiu Song, Min Zhu, Youxu Jiang, Shuhong Ma, Xiaofeng Zhuang, Shuzhen Guo

**Affiliations:** 1 School of Traditional Chinese Medicine, Beijing University of Chinese Medicine, Beijing, 100029 China; 2 Department of Nephrology, Beijing Hospital of Traditional Chinese Medicine, Capital Medical University, 23 Meishuguanhou Street, Dongcheng District, Beijing, 100010 China; 3 Beijing Laboratory for Cardiovascular Precision Medicine, The Key Laboratory of Biomedical Engineering for Cardiovascular Disease Research, Ministry of Education, Beijing Anzhen Hospital, Capital Medical University, Beijing, 100029 China; 4 Translational Medicine Collaborative Innovation Center, The Second Clinical Medical College (Shenzhen People’s Hospital), Jinan University, Shenzhen, 518020 China; 5 Post-Doctoral Scientific Research Station of Basic Medicine, Jinan University, Guangzhou, 510632 China; 6 Fuwai Hospital Chinese Academy of Medical Sciences, Shenzhen, 518057 Guangdong Province China; 7 Department of Cardiology, National Center for Cardiovascular Diseases, Chinese Academy of Medical Sciences and Peking Union Medical College, Fuwai Hospital, Beilishi Rd 167, Xicheng District, Beijing City, 100037 China; 8 Department of Emergency, The First Affiliated Hospital, Zhejiang University School of Medicine, Zhejiang, 310003 China; 9 Department of Cardiology, The Second Affiliated Hospital of Zhengzhou University, Jingba Road, Zhengzhou, 450053 China

**Keywords:** ADPRHL1, CRISPR/cas9, ROCK pathway, Focal adhesions

## Abstract

**Background:**

Pseudoenzymes, catalytically deficient variants of active enzymes, have a wide range of regulatory functions. ADP-ribosylhydrolase-like 1 (ADPRHL1), a pseudoenzyme belonging to a small group of ADP-ribosylhydrolase enzymes that lacks the amino acid residues necessary for catalytic activity, may have a significant role in heart development based on accumulating evidence. However, the specific function of ADPRHL1 in this process has not been elucidated. To investigate the role of ADPRHL1 in the heart, we generated the first in vitro human embryonic stem cell model with an ADPRHL1 knockout.

**Method:**

Using the CRISPR/Cas9 system, we generated ADPRHL1 knockout in the human embryonic stem cell (hESC) H9 line. The cells were differentiated into cardiomyocytes using a chemically defined and xeno-free method. We employed confocal laser microscopy to detect calcium transients and microelectrode array (MEA) to assess the electrophysiological activity of ADPRHL1 deficiency cardiomyocytes. Additionally, we investigated the cellular mechanism of ADPRHL1 by Bulk RNA sequencing and western blot.

**Results:**

The results indicate that the absence of ADPRHL1 in cardiomyocytes led to adhered abnormally, as well as perturbations in calcium transients and electrophysiological activity. We also revealed that disruption of focal adhesion formation in these cardiomyocytes was due to an excessive upregulation of the ROCK–myosin II pathway. Notably, inhibition of ROCK and myosin II effectively restores focal adhesions in ADPRHL1-deficient cardiomyocytes and improved electrical conduction and calcium activity.

**Conclusions:**

Our findings demonstrate that ADPRHL1 plays a critical role in maintaining the proper function of cardiomyocytes by regulating the ROCK–myosin II pathway, suggesting that it may serve as a potential drug target for the treatment of ADPRHL1-related diseases.

**Supplementary Information:**

The online version contains supplementary material available at 10.1186/s13287-023-03507-0.

## Introduction

Pseudoenzymes, which are catalytically deficient variants of active enzymes, are typically classified as atypical members of the enzyme superfamily [1]. Pseudoenzymes are ubiquitous in the proteome, accounting for approximately 10%–15% of the genome and found in more than twenty canonical superfamilies [2]. Despite their prevalence, pseudoenzymes were considered evolutionary vestiges without regulatory functions, and as a result, they have not received as much attention as their active protein counterparts. However, numerous recent investigations have revealed that pseudoenzymes play important role in regulating signaling and metabolic pathways [3–5]. Pseudoenzymes typically participate in the same signaling pathway as their ancestral enzyme and can function as either allosteric activators or inhibitors. Pseudoenzymes exhibit diverse modes of action, including (a) substrate-binding competitors that modulate the biological effects of active enzymes; (b) allosteric modulators of active enzymes; (c) steric anchors or substrate traps; and (d) signal modulators [6]. Given their diverse functions, functional deficiencies in pseudoenzymes are linked to numerous diseases, including cancer, obesity and neurological diseases [7–9]. Therefore, pseudoenzymes represent promising therapeutic targets for a broad range of diseases. Despite an increasing number of studies investigating pseudoenzymes and their functions in biological networks, many pseudoenzymes still have unknown functions [10]. Furthermore, the heart’s physiological functioning relies on complex signaling pathways, and little is currently known about the regulatory role of pseudoenzymes in this organ. ADPRHL1 belongs to the ADP-ribosylhydrolase (ARH) family in the vertebrate genome, which includes ADPRH, ADPRHL2 and ADPRHL3 [11, 12]. While the 354-amino acid sequence of human ADPRHL1 is 46% identical to ADPRH, which catalyzes the hydrolysis of mono-ADP-ribosyl groups from arginine residues of proteins in the ADP-ribosylation cycle, ADPRHL1 lacks the critical amino acids required for catalytic activity and is therefore considered a pseudoenzyme [13]. There is growing evidence that ADPRHL1 plays an essential role in heart development. Knockdown of ADPRHL1 in *Xenopus* embryos has been shown to cause severe heart defects [14], characterized by non-beating embryonic small ventricles and short and disorganized ventricular myofibrils. Various mutations in ADPRHL1 have been found to affect the development of the heart, despite its lack of catalytic activity, indicating the importance of its modified substrate-binding cleft [11]. Mutations in ADPRHL1 have been linked to significant defects in myofibril formation in ventricular cardiomyocytes (CMs). Studies have also demonstrated that the deletion of ADPRHL1 in chicken results in abnormal development of the heart, leg muscles, chest muscles and other tissues, providing further evidence of ADPRHL1’s essential role in vertebrate heart development [15]. In a clinical study conducted in Iceland, the missense variant p.Leu294Arg in ADPRHL1 was significantly associated with left anterior/posterior fascicular block (LAFB) [16]. These findings emphasize the critical role that ADPRHL1 plays in heart development and maintenance. Understanding how pseudoenzymes, such as ADPRHL1, regulate tissue development and function may offer valuable insights into their mechanisms of action.

To investigate the impact of ADPRHL1 on cardiac function, we employed the CRISPR/Cas9 system to generate an ADPRHL1-deficient human embryonic stem cell line, which was subsequently differentiated into ADPRHL1-deficient CMs. Our findings indicate that ADPRHL1 deficiency results in abnormal CMs adherence, and that the expressions of integrins and focal adhesions (FAs) are significantly reduced in these CMs as determined by western blot and immunofluorescence staining. RNA-seq and protein analyses suggest that the ROCK–myosin II pathway is impacted by ADPRHL1 deficiency, thereby influencing CM adhesion*.* We have demonstrated that treatment with a ROCK or myosin II inhibitor can effectively rescue the abnormal adhesion, electrophysiological and calcium changes observed in ADPRHL1-deficient CMs. To the best of our knowledge, this is the first time that a human model of ADPRHL1 deficiency has been established in vitro. Through this model, we have demonstrated that the pseudoenzyme ADPRHL1 plays a crucial role in CM adhesion by regulating the ROCK–myosin II pathway, and have identified a potential therapeutic target for ADPRHL-related diseases.

## Methods

### Cell culture and cardiac differentiation

The human embryonic stem cells line-H9 (hESCs-H9) was obtained from the Shanghai Institutes for Biological Sciences (18-1-1522, China) and cultured in PSCeasy medium (Cellapy, # CA1014500, China) at 37 °C with 5% CO2. The medium was changed daily, and cells were passaged when they reached 70–80% confluency using 0.5 mM EDTA (Cellapy, #CA3001500, China). After digestion, the cells were resuspended in PSCeasy medium and seeded in 6-well plates (Corning, USA) pre-coated with 5% Matrigel (Corning, #354,277, USA). To enhance cells survival, thiazovivin (Selleck Chemicals, #S1459, USA) was added to the PSCeasy medium at a concentration of 2 μM on the first day after passage.

Prior to differentiation, cells were tested for the absence of mycoplasma contamination. Cardiomyocyte differentiation was achieved by utilizing the Cardiomyocyte Differentiation Kit (Cellapy, #CA2004500, China). The components of the CardioEasy medium include RPMI 1640, 500 μg/mL Oryza sativa-derived recombinant human albumin (Sigma-Aldrich), as well as 213 μg/mL L-ascorbic acid 2-phosphate (Sigma-Aldrich,# 1,713,265–25-8), which is referred to as CDM3 medium. Briefly, at day 0, cells were washed twice with PBS (Hyclone, USA) and then cultured in CDM3 medium with supplemented 6μΜ CHIR99021 (Sigma, #SML1046, USA) and 25 ng/mL activin A for 2 days. For day 2 to day 4, medium was changed to CDM3 supplemented with 5 µM IWR-1(Selleck, #S7086). Subsequently, cells were maintained using CDM3 medium. Around 8 days following differentiation, cardiomyocytes exhibiting spontaneous beating could be observed. Highly purified CMs were accomplished using lactate metabolic selection.

### Genome editing

The single-stranded guide RNA “CCAATAAGTACCTTCTTCTCGCC” was designed using an online tool (http://crispr.mit.edu/) to target ADPRHL1. The resulting oligonucleotides were synthesized by Sino Geno Max (Beijing, China) and subsequently purified through desalination. The purified oligonucleotides were then ligated into the epiCRISPR vector, which contains both the puromycin resistance gene and copGFP driven by an EF1a promoter. This vector also includes the OriP/EBNA1 component, which enables replication under puromycin selection and thereby ensures sustained expression of both Cas9 and sgRNAs [17]. The resulting plasmids were then electroporated into H9 cells using the CA137 protocol with the 4D-Nucleofector system (Lonza, Germany). After 2 weeks of puromycin selection, surviving cell clones were sequenced to identify cell clones containing frameshift mutations in ADPRHL1.

### Immunofluorescence

Cells were fixed with 4% formaldehyde (Solarbio, #P1110, China) for 20 min at room temperature (RT). Subsequently, cells were permeabilized with 0.5% Triton X-100 (Invitrogen USA) for 20 min at RT. After blocking with 3% BSA for 30 min at RT, the cells were incubated with the following primary antibody: anti-SOX2 antibody (CST, #2748, USA), anti-SSEA4 antibody (CST, #4755, USA), anti-CX43 antibody (CST, #3512, USA), anti-Paxillin antibody (CST, #2542, USA) overnight at 4 °C. The following day, the cells were incubated at 37 °C for 1 h with secondary antibodies, which included Goat anti-Rabbit IgG Alexa Fluor 488(Invitrogen, # A-11008) and Goat anti-Mouse IgG Alexa Fluor 594(Invitrogen, # A-11005). Nuclei were stained with DAPI (Invitrogen, # D3571) for about 15 min at RT. Finally, images were captured using Leica DMI 400 fluorescence microscope and Leica TCS SP5 MP confocal laser scanning microscope (Leica, Germany).

### Flow cytometry

Cardiomyocytes were dissociated into single cells, fixed with 4% formaldehyde (Solarbio, China) for 20 min at RT, and permeabilized with 0.5% Triton X-100 (Invitrogen, USA) for 20 min at RT. After incubating with the primary antibody for 60 min, cells were washed with PBS for three times and then incubated with the secondary antibody for 30 min. Finally, the cells were immediately analyzed using a flow cytometry machine (BD Accuri C6, BD Bioscience, USA) and the data were analyzed using FlowJo V10 software.

### Western blot

Cells were dissociated and then pelleted by centrifugation at 1000 rpm/min for 5 min. The supernatant was removed, and the cell was lysed using Beyotime (# P0013B) lysis buffer. The lysate was vigorously shaken for 15 s and then placed on ice for 10 min. This step was repeated 3 times. After determining the protein concentration using the BCA method, 5X loading buffer was added, and the protein was denatured at 100 °C for 5 min. Electrophoresis was performed using 10% SDS-PAGE gels, and the samples were transferred to PVDF membranes at 300 mA for 90 min using a gel transfer device (Bio-Rad, USA). After blocking the PVDF membrane with 5% non-fat milk for 1 h at RT, the membrane was washed three times with TBST and then incubated with primary antibodies overnight at 4℃. The following day, the membranes were washed three times with TBST and then incubated with secondary antibodies, including Goat anti-Rabbit IgG(H + L) IRDye 800CW or Goat anti-mouse IgG(H + L) IRDye 800CW(LI-COR, USA) for 1 h at 37℃. Finally, the images were acquired using a UVA Bio imaging System (LI-COR Biosciences, USA).

### Quantitative reverse transcription PCR (qRT-PCR)

Cardiomyocytes were subjected to total RNA extraction using TRIzol Reagent (Invitrogen, USA), followed by DNase I (RNase free) (Beyotime, China) treatment. The concentration of RNA was measured using NANO drop 2000 (Thermo Fisher Scientific), and 1 µg total RNA was transcribed into cDNA using PrimeScript™ RT Master Mix (Takara, #RR036B, Japan). The gene expression levels were determined by Real-time PCR with TB Green™ Premix Ex Taq™ II (Takara, #RR820Q, Japan) on an iCycler iQ5 (Bio-Rad, USA). The relative changes in gene expression were analyzed using a comparative computed tomography method and normalized by the endogenous control gene *GAPDH*.

### Ca^2+^ imaging

Cardiomyocytes were cultured on Matrigel-coated confocal dishes and loaded with the calcium-sensitive fluorescent probe Fluo-4 (Beyotime, # S1061S, China). The intracellular calcium levels were monitored using a laser confocal microscopy (Leica, German) in line-scan mode. The acquired images were analyzed using Image J and Igor to detect changes in intracellular calcium levels.

### Microelectrode array (MEA) analysis

The human embryonic stem cell-derived cardiomyocytes (hESC-CMs) were digested using CardioEasy Human Cardiomyocyte Dissociation Kit (Cellapy, # CA2011100, China). Then, 2 × 10^4^ cells were plated on a microelectrode array (MEA) that was pre-coated with 5% Matrigel. The following day, 200μL of CDM3 medium was added to each well. The electrophysiology of the cardiomyocytes was recorded using a Maestro EDGE (Axion Biosystems, USA), and the data were analyzed using the Cardiac Analysis Tool, AxIS Navigator, AxIS data export tool and Origin. The conduction plot illustrates the propagation delay at each electrode from short (blue) to long (red). The beat originates in the blue region and terminates in the red. The more red there is, the slower the propagation speed between the two electrodes, which can be used to indicate signal conduction velocity.

### RNA sequencing (RNA-seq)

Clustering and sequencing were performed by Beijing Genomics institution (BGI, China). The screening of differential genes is based mainly on the differential multiple (fold change value) and q value (p adjusted value, corrected p value). The screening criteria of significantly different genes were |log2 fold change|R1 and q < 0.05. All differential genes were analyzed by hierarchical cluster analysis using R-package, and heatmaps were drawn. Gene Ontology (GO) counts differential genes and finds significantly enriched GO items according to the standard of q < 0.05. The enrichment pathway analysis of differential genes was performed through the Kyoto Encyclopedia of Genes and Genomes (KEGG) database.

### Statistical analysis

All experiments were conducted at least 3 times. All data are displayed as the mean ± standard error of mean. GraphPad Prism 8 was used for statistical analysis which was carried. Two-sided unpaired Student’s *t* test was used to compare two groups with normal distribution, and one-way ANOVA was used to compare three or more groups. Normality and homogeneity of variance were conducted prior to t test and one-way ANOVA. A *p* value less than 0.05 was considered statistically significant.

## Result

### ADPRHL1 deficiency does not affect CM differentiation, but disrupts CM adhesion

**Fig. 1:**
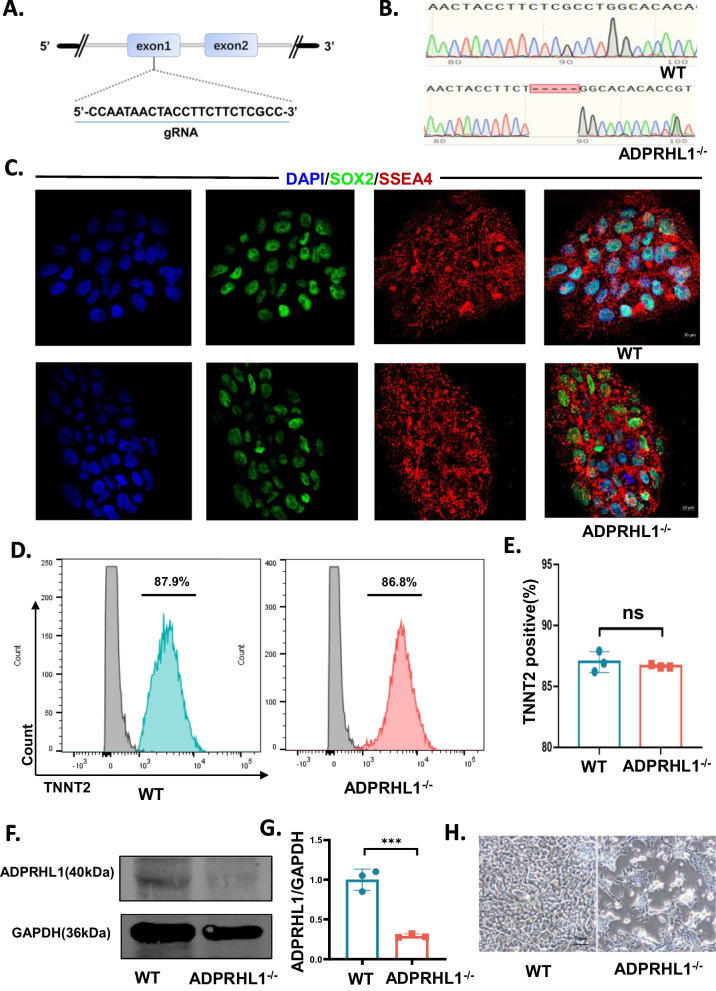
ADPRHL1 deficiency does not affect CM differentiation, but disrupts CM adhesion. **A** Structure of the ADPRHL1 locus showing localization of gRNA for epiCRISPR/Cas9 editing. **B**) Sequence chromatograms demonstrate 5-base insertion in ADPRHL1 knockout (KO) colonies. **C** Immunofluorescence staining of KO colonies for the pluripotency markers SOX2 and SSEA4. Scale bar, 10 μm. **D** and **E** Flow cytometry analysis for TNNT2 from representative WT and KO differentiation protocols before purification at day 15. **F** Western blot analysis of ADPRHL1 in WT-CMs and KO-CMs at day 15; **G** Quantification of ADPRHL1 normalized by GAPDH in WT-CMs and KO-CMs. **H** Adherence of WT-CMs and KO-CMs after re-seeding on day 15. Scale bar, 50 μm. The results are presented as means ± SEMs of 3 independent experiments. ns, not significant, unpaired 2-sided Student’s t test

To generate the ADPRHL1 knockout hESCs-H9 (KO), we employed a highly efficient epiCRISPR /Cas9 gene-editing system [17] to target exon 1 of ADPRHL1 (Fig. 1A). The vector was electroporated into H9. After puromycin selection, we isolated and expanded single-cell-derived clones for genotyping and found one homozygous ADPRHL1 knockout clone. This clone exhibited a 5-base-pair deletion resulting a frame-shifted coding sequence with a premature stop codon (Fig. 1B). Despite lacking ADPRHL1, the KO displayed normal morphology and expressed pluripotency markers SOX2 and SSEA4 (Fig. 1C and Additional file 1: S1C-S1E). To investigate the effect of ADPRHL1 deficiency on cardiac development, we differentiated the KO and the parental hESCs-H9 (WT) cells toward CM using a chemically defined and xeno-free method [18] (Additional file 1: Figure S1A). Before differentiation, we confirmed that both WT and KO cells were free of mycoplasma (Additional file 1: Figure S1B). On the second day, we detected the expression levels of the mesoderm markers Mesp1 and DKK1 using qRT-PCR analysis (Additional file 1: Figure S1F and S1G), and the results showed no significant difference between WT and KO cells. On the fourth day, the detection of the cardiac precursor markers TBXT and NKX2.5 also showed no statistically significant difference between the two groups (Additional file 1: Figure S1H, I). After ten days, both WT and KO cells successfully differentiated into CMs that could spontaneously beat. Flow cytometry showed that both WT-CM and KO-CM expressed cardiac troponin T (cTNT) on fifteenth day, with no statistically significant difference between them (*P* = 0.5398), indicating that ADPRHL1 deficiency does not influence the ability to generate CMs in vitro (Fig. 1D, E). qRT-PCR results also showed no difference in the expression of heart specific genes between WT-CM and KO-CM (Additional file 1: Figure S1J). However, there were certain differences observed in the expression of ventricular and atrial markers (Additional file 1: Figure S2A-C). The immunofluorescence results confirmed the expression of sarcomere-specific markers α-actinin and cTnT in both the WT and KO groups. In WT-CM, distinct cross-striations and well-arranged sarcomeres were parallel to the cell’s long axis. However, in KO cardiomyocytes, the arrangement of sarcomeres was less regular (Additional file 1: Figure S2D). Moreover, western blot confirmed the absence of ADPRHL1 in KO-CM (Fig. 1F, G). Interestingly, in culture, the KO-CM showed large areas of detachment from the culture plate. We seeded an equal number of WT-CMs and KO-CMs onto a cell culture plate after 5–10 days of spontaneous beating. To investigate this phenomenon further, we seeded an equal number of WT-CM and KO-CM onto a cell culture plate. In our result, as shown in Fig. 1H, the majority of WT-CM adhered to the surface of the culture plate, while only a portion of KO-CM displayed adhesion, suggesting that the lack of ADPRHL1 may disrupt cell-extracellular matrix (ECM) adhesion.

### ADPRHL1 deficiency decreases FA formation

**Fig. 2:**
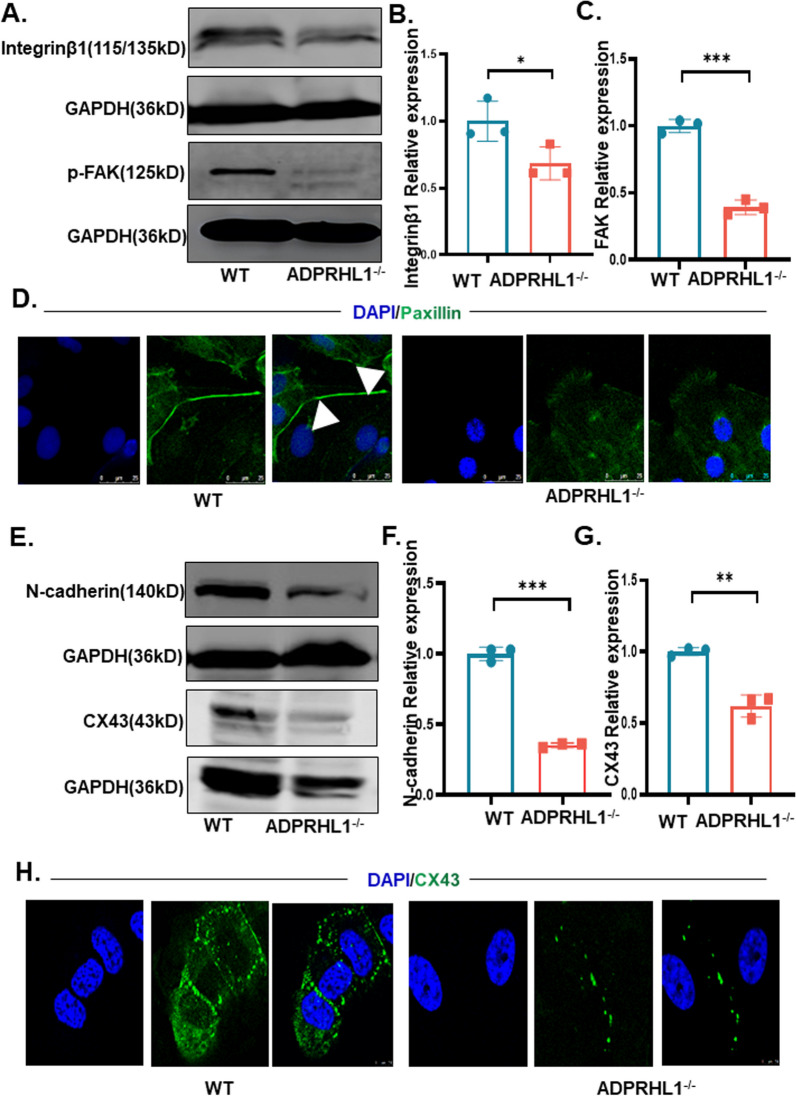
ADPRHL deficiency decreases FA formation. **A** Western blot analysis of β1-integrin and p-FAK in WT-CMs and KO-CMs at day 20. **B** and **C** Quantification of β1-integrin and FAK normalized by GAPDH in WT-CMs and KO-CMs at day 20. **D** Immunofluorescence staining of WT-CMs and KO-CMs for paxillin. Scale bar, 25 μm. **E **Western blot analysis of N-cadherin and Cx43 in WT-CMs and KO-CMs at day 20. **F** and **G** Quantification of N-cadherin and Cx43 in WT-CMs and KO-CMs at day 20. **H** Immunofluorescence staining of WT-CMs and KO-CMs for Cx43. Scale bar, 7.5 μm. Results are presented as means ± S.E.M. of three independent experiments. *P < 0.05; **P < 0.01; ***P < 0.001; unpaired two-sided Student’s t test

The ECM cell extracellular matrix (ECM) and the cell are intimately connected through focal adhesions (FAs), which rely on integrins as their primary adhesion molecules [19, 20]. The FAs consist of two classes of proteins. The first is the “scaffolding molecules.” The second is a variety of regulatory proteins including tyrosine-specific and serine/threonine-specific protein kinases and phosphatases [21]. Previous studies have demonstrated that integrins are the main components of the scaffolding molecules of FAs and play a central role in the formation of matrix protein receptors [22]. Integrins are heterodimeric surface receptors consisting of a larger α subunit and a smaller β subunit. The integrin that binds primarily to ECM is the αβ1-integrin [23]. We investigated the expression of β1-integrin in KO-CM. To accomplish this, we extracted proteins from WT-CM and KO-CM for western blotting analysis. Our results indicate that the expression of β1-integrin was significantly decreased in KO-CM, suggesting that ADPRHL1 deficiency disrupts the FAs in these cells (Fig. 2A, B). Moreover, we found that phosphorylated focal adhesion kinase (FAK) was significantly downregulated in KO-CM (Fig. 2A, C), indicating that the regulation of FA-related signaling pathways may be perturbed in KO-CMs. Furthermore, we found that the expression of Paxillin, a structural protein in FAs, in KO-CM was markedly reduced by immunofluorescence staining and qRT-PCR analyses (Fig. 2D and Additional file 1: Figure S2E), suggesting that defect in FA formation may underlie the abnormal adhesion of KO-CM. In additionally, Talin (TLN1) function is critical for cell-ECM adhesion and is required for integrin adhesion complex assembly and maintenance [24], and the expression of TLN1 gene was significantly downregulated in KO-CM analyzed by qRT-PCR (Additional file 1: Figure S2F). Because the N-cadherin plays a crucial role in cell-ECM adhesion, it links to actin filaments, allowing the cell to anchor to the ECM and form stable focal adhesions [25, 26]. Additionally, N-cadherin mediates strong homophilic cell–cell adhesion by linking to the actin cytoskeleton [27]. Therefore, we detected the expression of N-cadherin by western blotting analysis, and the results showed that the expression of N-cadherin was significantly downregulated in KO-CM compared with WT-CM (Fig. 2E, F). Western blot and immunofluorescence staining analyses also revealed that the expression of Cx43 in KO-CM was decreased, indicating that gap junctions in these cells were also affected (Fig. 2E, G, H). Furthermore, vinculin (VCL) as a cytoskeletal protein was related to the cytoplasmic face of both cell–cell and cell-extracellular matrix adherens-type junctions. It is thought to function as one of several interacting proteins involved in anchoring F-actin to the membrane, providing the basis for network formation within FA [28]; the expression of VCL gene was downregulated in KO-CM (Additional file 1: Figure S2G). Overall, our findings suggest that the destruction of FAs caused by ADPRHL1 deficiency directly leads to adhesion disorders and indirectly affects the intercellular adhesion of CMs.

### Adhesion disorder affects electrical conduction and calcium transients in CMs

**Fig. 3:**
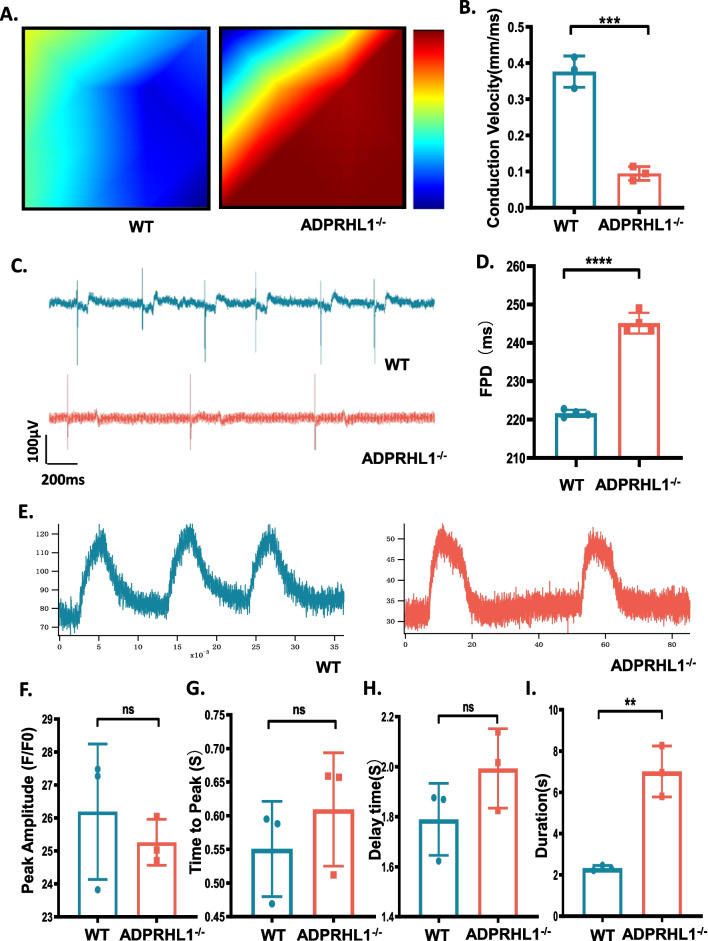
Adhesion disorder affects electrical conduction and calcium transients in CMs. **A** and **B** MEA detection of the electrical conduction velocity of WT-CMs and KO-CMs at day 20. **C** and **D** MEA detection of the FPD of WT-CMs and KO-CMs at day 20. **E** Representative line-scan images in WT-CMs and KO-CMs at days 20. **F**, **G**, **H** and **I** Quantification of peak, time to peak, delay time and calcium transient duration in WT-CMs and KO-CMs. Results are presented as means ± S.E.M. of three independent experiments. **P < 0.01; ***P < 0.001; ****P < 0.0001; ns, not significant, unpaired two-sided Student’s t test

Impairment of cell–cell adhesion by ADPRHL1 deficiency may lead to abnormal electrical conduction in KO-CM, as intercellular gap junctions are critical for electrical signal conduction. To investigate this, we examined the electrophysiology of KO-CM using a microelectrode array (MEA). The conduction plot illustrates the propagation delay at each electrode from short (blue) to long (red). The beat originates in the blue region and terminates in the red, and the more red there is, the slower the propagation speed between the two electrodes, which can be used to indicate signal conduction velocity. Our results showed that the electrical conduction in KO-CM was significantly slowed (Fig. 3A, B). Field potential analysis showed that the frequency of action potentials in KO-CM was decreased, and the field potential duration (FPD) was prolonged (Fig. 3C, D). During the excitation–contraction coupling process, excitation regulates contraction by affecting the release of calcium in CM [29]. Therefore, electrophysiological abnormalities may also affect calcium activity in KO-CM. To investigate this, we used the fluorescent indicator Fluo-4 to monitor calcium transients in KO-CM (Fig. 3E). We found that there was no significant difference in peak amplitude between WT-CM and KO-CM (Fig. 3F). The time to peak and delay time were slightly longer in KO-CM, but the difference was not significant (Fig. 3G, H). However, the duration of the calcium transient in KO-CM was significantly prolonged, consistent with the electrophysiological phenotype (Fig. 3I). Taken together, these results show that the failure in adhesion caused by ADPRHL1 deficiency results in abnormal myocardial electrophysiology and calcium activity, suggesting that ADPRHL1 mutation in vivo is likely to affect the cardiac electrophysiological activity.

### Transcriptional profiling suggests that ADPRHL1 deficiency disrupts cell adhesion by activating the ROCK–myosin II pathway

**Fig. 4:**
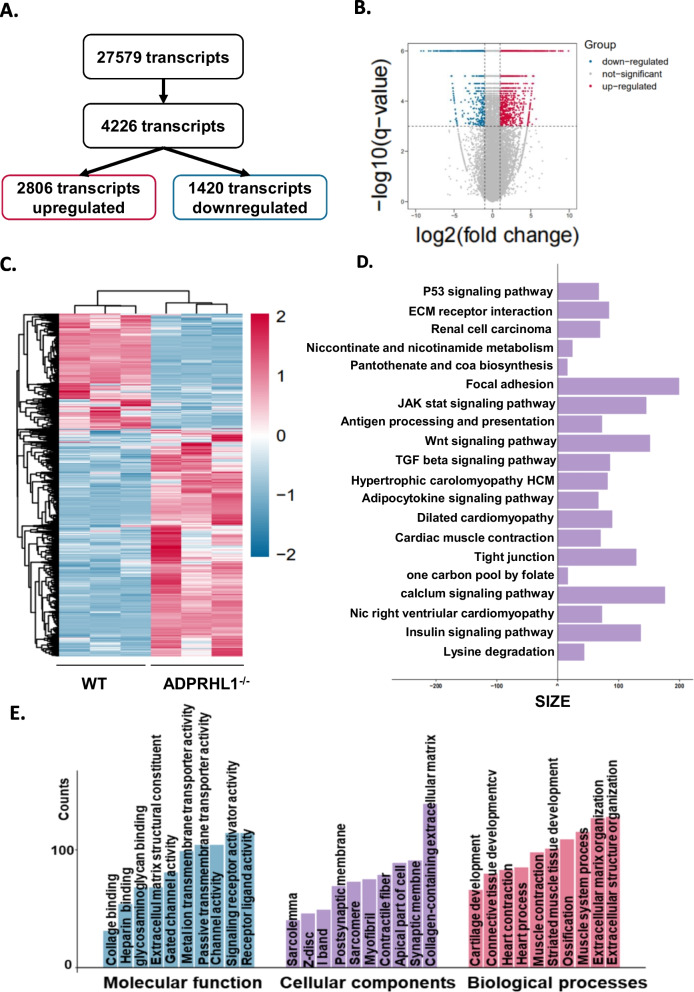
Transcriptional profiling of ADPRHL1-deficient CMs. **A** Analysis of significant differential genes of WT-CMs and KO-CMs at day 20 (FDR < 0.001, log2FC ± 1). **B** Volcano plot showed the distribution of differential genes of WT-CMs and KO-CMs at day 20. **C **Gene clustering by Z score using Euclidean distance metric in WT-CMs and KO-CMs at day 20. **D** Enriched KEGG pathway analysis showed the significantly enriched pathways of WT-CMs and KO-CMs at day 20. **E** GO analysis showed the DEGs of WT-CMs and KO-CMs at day 20 were enriched in cellular components, molecular function and biological processes

To elucidate the mechanism by which ADPRHL1 deficiency disrupts the formation of FAs, we performed global transcriptome analysis on WT-CM and KO-CM collected within 3 days after re-seeding. We identified a total of 4,226 significant differential genes (DEGs) using stringent threshold criteria (false discovery rate (FDR) < 0.001, log2FC ± 1), with 2,806 upregulated genes and 1,420 downregulated genes (Fig. 4A, B). The heatmap analysis showed excellent consistency of differential transcripts within groups (Fig. 4C). Enriched KEGG pathway analysis revealed that the pathways regulating focal adhesion, ECM receptor interaction, tight junctions and cardiac muscle contraction were significantly enriched (Fig. 4D). GO analysis revealed which categories within cellular components, molecular function and biological processes that were enriched for DEGs (Fig. 4E). Several GO terms, such as cell adhesion, extracellular matrix organization and extracellular matrix structural constituent, were intimately related to ADPRHL1 deficiency. Given that ADPRHL1 deficiency leads to the failure of KO-CM to adhere by disrupting FAs, we focused on the downstream FA pathways. And among the downstream FA pathways, sequencing results showed abnormal expression of ROCK–myosin II pathway and are closely related to cell adhesion [30, 31], suggesting that it may play an important role in the adhesion disorder in ADPRHL1-deficient CMs.

### Inhibition of the ROCK–myosin II pathway rescues the cell adhesion phenotype in ADPRHL1-deficient cardiomyocytes

**Fig. 5:**
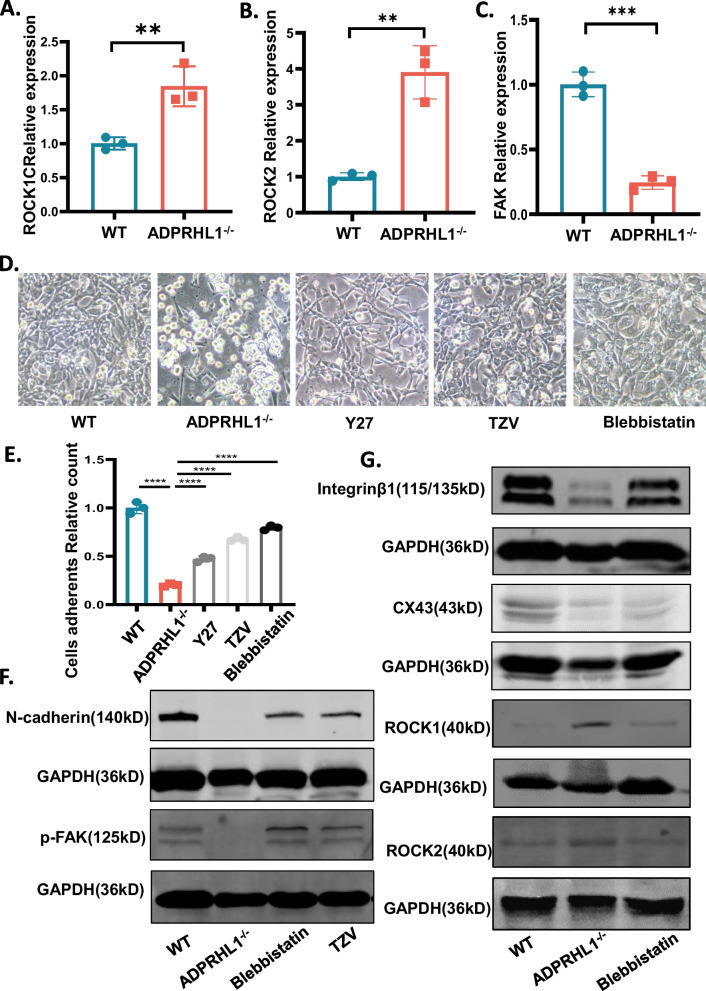
Inhibition of the ROCK pathway rescues the cell adhesion phenotype of KO-CMs. (**A**, **B** and **C**) Quantification q-PCR analysis ROCK1, ROCK2 and FAK normalized by GAPDH in WT-CMs and KO-CMs at day 20. (**D**) Adherence of WT-CMs, KO-CMs and KO-CMs with drug treatment after re-seeding on day 15. (**E**) Quantification of cells adherent relative count. (**F**) Western blot analysis of N-cadherin and p-FAK in WT-CMs, KO-CMs and KO-CMs treated with Blebbistatin and TZV at day 20. (**G**) Western blot analysis of Integrin-β1, Cx43, ROCK1 and ROCK2 in WT-CMs, KO-CMs and KO-CMs treated with Blebbistatin at day 20.Results are presented as means ± S.E.M. of three independent experiments. **P < 0.01; ***P < 0.001; unpaired two-sided Student’s t test

To investigate the impact of ADPRHL1 deficiency on cell adhesion via the ROCK pathway, we conducted an expression of ROCK in KO-CM. Our results from qRT-PCR showed that KO-CMs exhibit significantly increased expression of both ROCK1 and ROCK2 (Fig. 5A, B), as well as significantly decreased expression of FAK (Fig. 5C). Previous studies have demonstrated that excessive upregulation of ROCK results in the overactivation of stress fibers, leading to the destruction of FAs [20, 32]. To further investigate the involvement of the ROCK–myosin II pathway in the abnormal cell adhesion of KO-CM, we treated these cells with Y-27632, a selective ROCK1 inhibitor, Thiazovivin, a novel ROCK inhibitor, and Blebbistatin, a selective inhibitor of myosin II [33–35]. Our results indicate that after drug treatment, KO-CMs were able to adhere normally, indicating that the cell adhesion disorder caused by ADPRHL1 deficiency is indeed related to the ROCK–myosin II pathway (Fig. 5D, E). Among the drugs tested, the expression levels of p-FAK and N-cadherin returned to normal levels in KO-CMs after Blebbistatin and Thiazovivin treatment, indicating that inhibition of the ROCK–myosin II pathway rescues the cell–ECM and cell–cell adhesion defects caused by ADPRHL1 deficiency (Fig. 5F). Immunofluorescence staining for paxillin, returned to normal levels in KO-CMs after Blebbistatin treatment, demonstrated that FAs had reformed in these cells (Additional file 1: Figure S3A). In additionally, Blebbistatin was found to be the most effectively at restoring adhesion of KO-CMs, as evidenced by the restoration of ROCK expression to normal levels (Fig. 5G). Furthermore, the expression levels of β1-integrin returned to normal levels in KO-CMs after Blebbistatin treatment (Fig. 5G). The expression levels of Cx43 in KO-CMs returned to normal levels, indicating that intercellular adhesion among KO-CMs had also improved (Fig. 5G). These results were further confirmed by qRT-PCR analysis (Additional file 1: Figure S3B-S3F), which indicates that inhibition of the ROCK–myosin II pathway rescues the cell–ECM and cell–cell adhesion defects caused by ADPRHL1 deficiency.

### Blebbistatin improves electrical conduction and calcium transients in ADPRHL1-deficient CMs

**Fig. 6:**
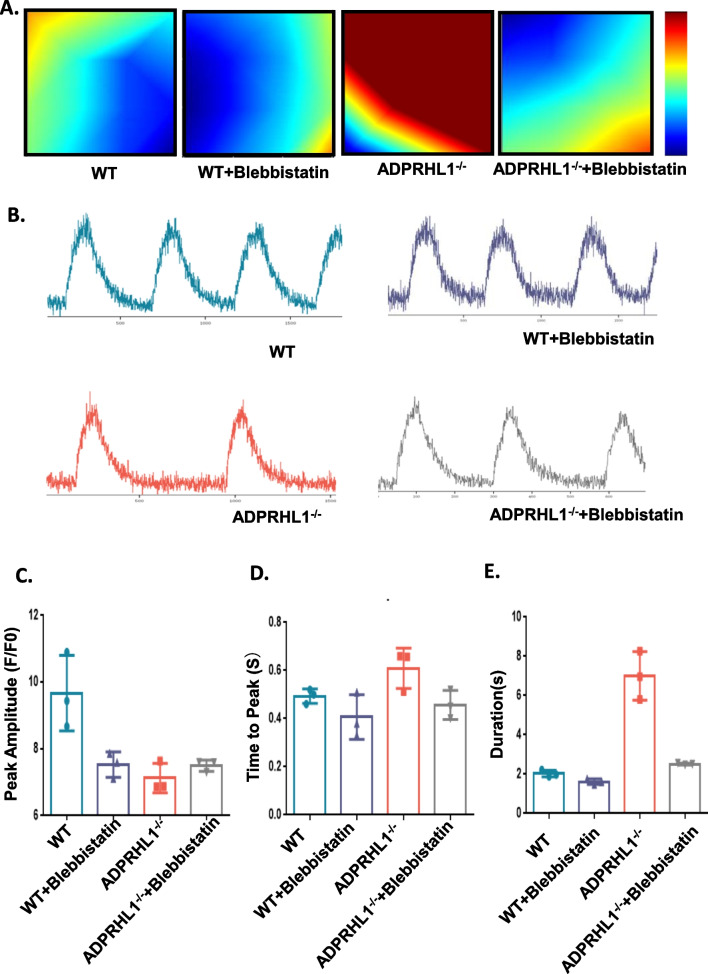
Blebbistatin improves electrical conduction and calcium transients of KO-CMs. **A** MEA detection of the electrical conduction velocity of WT-CMs, KO-CMs, WT-CMs treated with Blebbistatin and KO-CMs treated with Blebbistatin at day 20. **B** Representative line-scan images in WT-CMs, KO-CMs, WT-CMs treated with Blebbistatin and KO-CMs treated with Blebbistatin at day 20. **C**, **D** and **E** Quantification of peak, time to peak and calcium transient duration in WT-CMs, KO-CMs, WT-CMs treated with Blebbistatin and KO-CMs treated with Blebbistatin

To determine whether restoration of abnormal cell adhesion in KO-CMs, achieved by inhibiting the ROCK-myosin II pathway, can improve electrophysiological and calcium transient phenotypes, we analyzed the electrophysiological and calcium transient properties of the cells after Blebbistatin treatment. Our results, presented in Fig. 6A, Additional file 1: Fig. S4A and S4B, demonstrate that both electrical conduction and action potential frequency in the CMs were normalized after Blebbistatin treatment. Furthermore, Blebbistatin treatment significantly reduced the duration of the abnormally prolonged calcium transient in the KO-CMs (Fig. 6B–E).

## Discussion

Pseudoenzymes are a group of proteins that have undergone evolutionary mutations resulting in the loss of their catalytic activity [36]. Historically, these proteins were considered evolutionary remnants with no physiological function due to their lack of catalytic activity. However, recent studies have shown that pseudoenzymes play important roles in signal transduction and metabolic regulatory [3]. Pseudoenzymes can act as allosteric regulators, signal integrators, nucleators of protein complex assembly or as competitors for substrate binding with conventional enzymes [6, 8]. Several diseases, such as cancer, obesity and neurological disorders, have been linked to pseudoenzymes [7–9]. Therefore, investigating the functions of pseudoenzymes could lead to the discovery of new therapeutic targets for a variety of diseases [36]. Despite the fact that the regulation of complex signaling pathways is essential for normal cardiac function, the role of pseudoenzymes in the heart remains largely unknown. To address this knowledge gap, we focused on the pseudoenzyme ADPRHL1, which has been reported to play a role in cardiac development.

ADPRHL1 is a member of the ARH protein family [37, 38], which is involved in post-translational modification of proteins through ADP ribosylation. ADP-ribosylation is catalyzed by ADP-ribosyltransferase, and ARH family members, including ADPRHL1, can restore the function of target protein by hydrolyzing the ADP-ribose moiety [39–42]. Despite lacking the necessary amino acids required for catalytic activity, ADPRHL1 has been shown to play a significant role in heart development and function. Studies using *Xenopus* embryos have demonstrated that ADPRHL1 deficiency leads to abnormal cardiac cavity formation, indicating its essential role in cardiac development, even in the absence of catalytic activity in its substrate-binding cleft [11, 14]. In a clinical study, mutations in ADPRHL1 were found to be significantly associated with left anterior fascicular block (LAFB), which further suggests its role in human cardiac function [16]. Although the exact mechanism of ADPRHL1 in the heart remains unknown, these findings strongly suggest that ADPRHL1 acts as a regulatory factor in cardiac function as a pseudoenzyme.

The present study aimed to investigate the regulatory function and mechanism of ADPRHL1 in the heart. To achieve this, ADPRHL1 was knocked out in human embryonic stem cells using CRISPR/Cas9 technology, and the cells were differentiated into cardiomyocytes to produce a human myocardial model of ADPRHL1 deficiency. This is the first in vitro model of ADPRHL1 deficiency in humans and is expected to better reflect the functional impact of ADPRHL1 deficiency on cardiomyocytes at the cellular level compared to in vivo models. The study found that the adhesion of ADPRHL1-deficient cardiomyocytes was severely disrupted. This abnormal adhesion phenotype is difficult to observe in animal models, indicating the advantages of cellular models [43–45]. Compared with in vivo experiments, in vitro experiments also have the disadvantage of lacking complexity as well as interaction of living organisms; therefore, the results could not accurately represent the actual physiological response in vivo, and there are weaknesses in extrapolating to the human. Therefore, this study is expected to in-depth in vivo experiments in the future [46]. The expression of β1-integrin and N-cadherin in KO-CMs was reduced, resulting in disruption of both cell–ECM and cell–cell adhesion in KO-CMs. Based on transcriptional profiling, the study speculated that ADPRHL1 deficiency results in abnormal cardiomyocyte adhesion through upregulation of the ROCK pathway. In our study, we observed a significant upregulation of ROCK in KO-CMs. It is known that under normal physiological conditions, ROCK upregulation can increase cardiomyocyte contraction force, which promote the formation of FAs between cell-surface integrins and the ECM. After the formation of FAs, the ROCK pathway returns to normal activity levels. However, excessive upregulation of ROCK can cause excessive contraction of stress fibers [20], resulting in cells failing to adhere. In the absence of ADPRHL1, we found that ROCK activity was significantly upregulated leading to excessive contraction of stress fiber and the perturbation of N-cadherin complexes, resulting in a vicious positive feedback loop [47]. Treatment with ROCK and myosin II inhibitors downregulated ROCK in KO-CMs, which restored their ability to adhere, highlighting the role of ROCK in this process. Our findings suggest that the excessive upregulation of ROCK caused by the absence of ADPRHL1 is the primary cause of cardiomyocytes adhesion disruption. ADPRHL1 belongs to the ARH family, which is responsible for hydrolyzing the ADP moiety in modified proteins and restoring protein activity [12]. Pseudoenzymes typically function in the same pathway as their ancestral active enzymes, suggesting that ADPRHL1 may be a competitive inhibitor of ARH. We speculate that after ROCK undergoes ADP ribosylation, ADPRHL1 may prevent ARH from hydrolyzing ADP through competitive binding, thereby inhibiting the activity of ROCK. Conversely, in the absence of ADPRHL1, ROCK activity cannot be inhibited, leading to excessive contraction and a vicious positive feedback cycle.

Previous studies on ADPRHL1 have not proposed a similar mechanism, but our findings of a vicious positive feedback loop of excessive ROCK upregulation due to ADPRHL1 deficiency can provide an explanation for the observed phenotypes. The excessive ROCK upregulation leads to the excessive contraction of myofibrils, which leads to the loss of their structural integrity, providing an explanation for the observed myofibril disorder in *Xenopus* embryos [14]. Moreover, the excessive ROCK activity leads to the failure to form functional FAs, which are essential for myocardium migration, thereby explaining the developmental arrest of the Xenopus heart caused by the loss of ADPRHL1. Additionally, our study observed a reduction of cellular connections and a slowing of cell-to-cell conduction in KO-CMs, which are consistent with the pathological phenotype observed in patients with the ADPRHL1 p.Leu294Arg variant found by genome-wide diagnosis. This variant is significantly correlated with LAFB conduction defects, and the mutation is located in exon 6 of ADPRHL1, which is conserved in vertebrates. The mutation is situated in the α-helix below the active site. Our study’s proposed mechanism of action of ADPRHL1 can explain the phenotype observed in the previous studies, including the reduced Cx43 levels and the cell conduction phenotype observed in this study.

Furthermore, electrophysiology and calcium transient analyses were performed to evaluate the physiological activity of KO-CMs. The results showed that ADPRHL1 deficiency also contributes to abnormal physiological activity in the heart, as evidenced by conduction slowing and abnormal calcium activity. This suggests that the impact of ADPRHL1 deficiency on the heart is not limited to development, but also affects the normal physiological activities of the heart, potentially leading to heart disease. Interestingly, treatment of KO-CMs with the ROCK inhibitor TZV and the myosin II inhibitor Blebbistatin resulted in the restoration of cardiomyocyte adhesion and cell–cell adhesive interactions. Our finding also suggests that inhibition of the ROCK–myosin II pathway or direct inhibition of myofibril contraction can block the vicious positive feedback loop induced by ADPRHL1 deficiency, providing potential therapeutic strategies for ADPRHL1-related diseases.

**Fig. 7:**
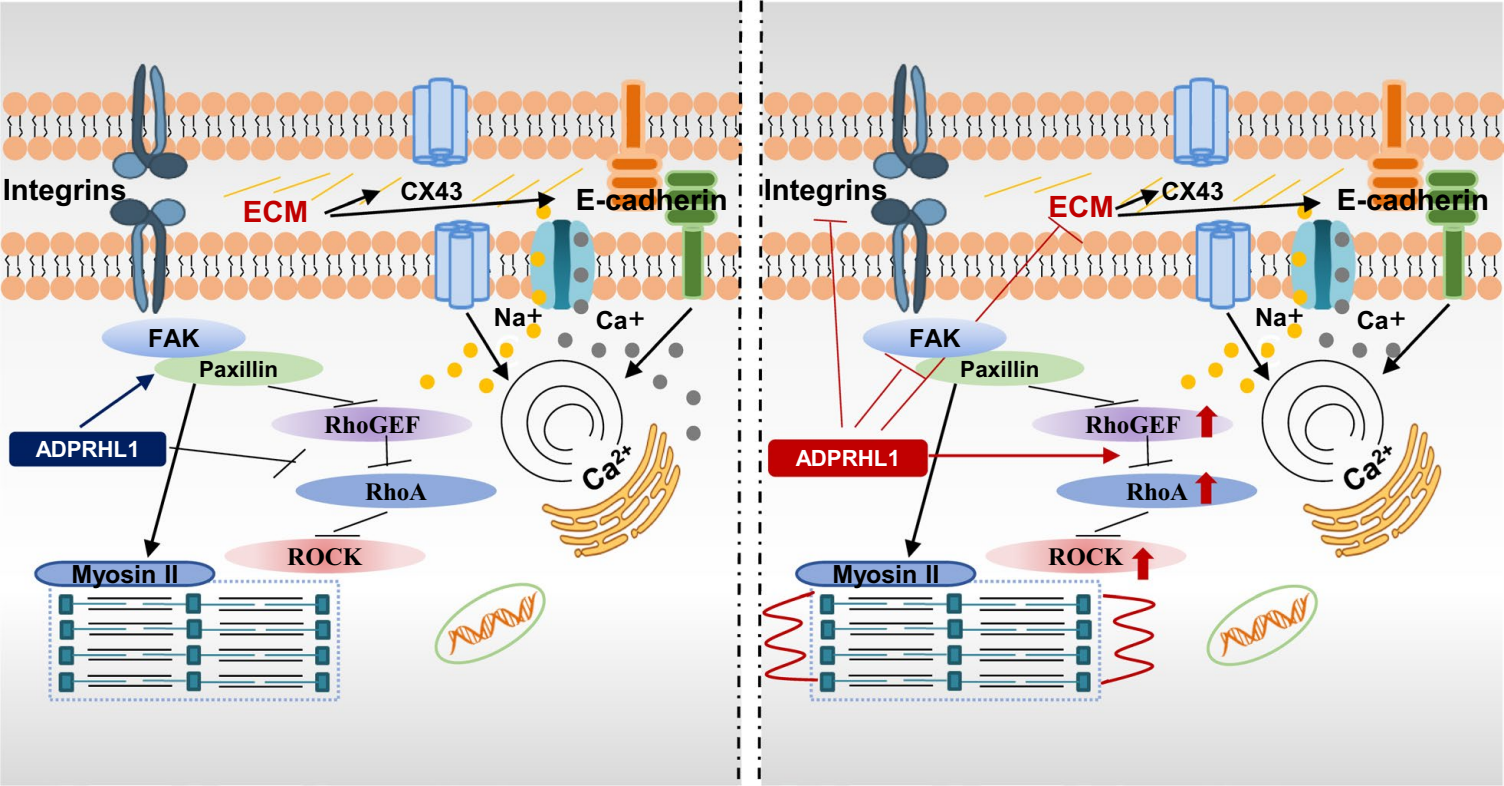
Schematic illustrating the mechanism of cardiomyocyte injury after ADPRHL1 gene knockout

In summary, this study reports the development of an ADPRHL1-deficient human cardiomyocyte model in vitro for the first time. The findings suggest that ADPRHL1 promotes cardiomyocyte adhesion by inhibiting excessive activation of the ROCK pathway. Deficiency in ADPRHL1 function may lead to excessive upregulation of ROCK, disrupting of cell–ECM and cell–cell adhesive interactions. In vivo, the vicious positive feedback loop may cause conduction block, impaired myocardial migration and structural disruption of myofibers during myocardial development, ultimately leading to abnormal cardiac development and function. ROCK and myosin II inhibitors effectively interrupt this process, restoring myocardial adhesion and intercellular connections, and rescuing other abnormal phenotypes in cardiomyocytes (Fig. 7). These findings expand our understanding of pseudoenzymes in cardiac regulation and provide a new direction for functional studies of pseudoenzymes.

## Conclusions

In this study, we established an in vitro ADPRHL1 knockout human myocardial model using the CRISPR/Cas9 system and investigate the function of ADPRHL1 in cardiomyocytes. Our results demonstrated that ADPRHL1-deficient cardiomyocytes exhibited abnormal adhesion, calcium transients and electrophysiological activity. Further experiments revealed that ADPRHL1 deficiency disrupted the formation of focal adhesions in cardiomyocytes by excessively upregulating the ROCK–myosin II pathway. Treatment with inhibitors of ROCK and myosin II effectively restored the focal adhesions of ADPRHL1-deficient cardiomyocytes and improved electrical conduction and calcium activity. These findings provide new insights into the role of ADPRHL1 as a pseudoenzyme in the heart and offer a potential therapeutic target for its related diseases.

### Supplementary Information

**Additional file 1.** **Supplementary figure 1.** Identification of pluripotency markers and cardiomyocyte markers. (**A**) Schematic diagram of the CM differentiation method. (**B**) The WT and KO cells were tested for mycoplasma.(**C**, **D** and **E**) Quantification q-PCR analysis of SOX2, OCT4 and NANOG normalized by GAPDH in WT and KO at day 20. (**F** and **G**) Quantitative q-PCR analysis of Mesp1 and DKK1 normalized by GAPDH in WT and KO cells at day 2. (**H**, **I** and **J**) Quantitative q-PCR analysis of TBXT, NKX2.5 and TNNT2 normalized by GAPDH in WT and KO cells at day 4. **Supplementary figure 2.** Cardiomyocyte morphology and adhesion related indicators. (**A**, **B** and **C**) Transcriptional profiling data of GATA4, MYH6 and MYH7. (**D**) Immunofluorescence staining of WT-CMs and KO-CMs for α-actinin and cTNT. Scale bar, 20 μm. (**E**, **F** and **G**) Quantitative q-PCR analysis of Paxillin, TLN1 and VCL. **Supplementary figure 3.** Inhibition of the ROCK pathway rescues the cell adhesion phenotype of KO-CMs. (**A**) Immunofluorescence staining of WT-CMs, KO-CMs, and KO-CMs treated with Blebbistatin for paxillin. Scale bar, 10 μm (**B**) Quantitative q-PCR analysis of ROCK1, ROCK2, FAK, PXN, ITGB1 and Cx43 normalized by GAPDH in WT-CMs, KO-CMs and KO-CMs treated with Blebbistatin at day 20. **Supplementary figure 4.** Blebbistatin improves electrical conduction and calcium transients of KO-CMs. (**A** and **B**) MEA detection of the field potential duration of WT-CMs, KO-CMs, WT-CMs treated with Blebbistatin and KO-CMs treated with Blebbistatin at day 20, (n = 3 cells per group).

## Data Availability

We uploaded the RNA-seq raw data to the National Center for Biotechnology Information (NCBI) archives series https://www.ncbi.nlm.nih.gov/geo/query/acc.cgi?acc=GSE235522.

## Abbreviations

ADPRHL1: ADP-ribosylhydrolase-like 1
hESC: Human embryonic stem cell
CMs: Cardiomyocytes
LAFB: Left anterior/posterior fascicular block
FAs: Focal adhesions
VCL: Vinculin
TLN1: Talin
MEA: Microelectrode array
KO: Knockout
FPD: Field potential duration
FDR: False discovery rate
GSEA: Gene Set Enrichment Analysis
GO: Gene Ontology
ECM: Cell-extracellular matrix
RNA-seq: RNA sequencing
KEGG: Kyoto Encyclopedia of Genes and Genomes
DEGs: Differentially expressed genes
