# Supplementary material for: The pseudoenzyme ADPRHL1 affects cardiac function by regulating the ROCK pathway
Source: Stem Cell Res Ther. 2023 Oct 26;14:309. doi: 10.1186/s13287-023-03507-0 (PMC10601310; doi:10.1186/s13287-023-03507-0)

Supplementary figure 1

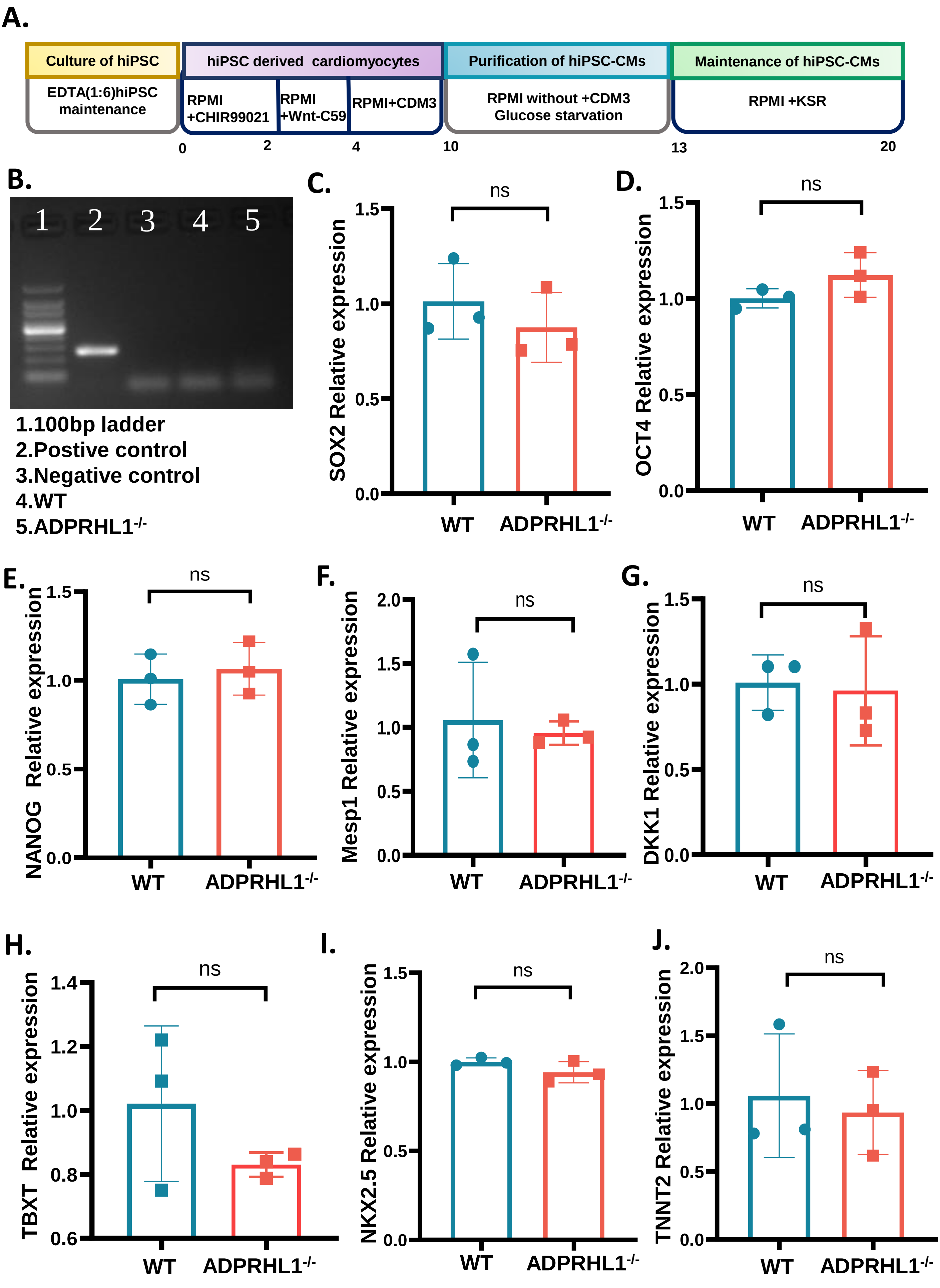

Supplementary figure 2

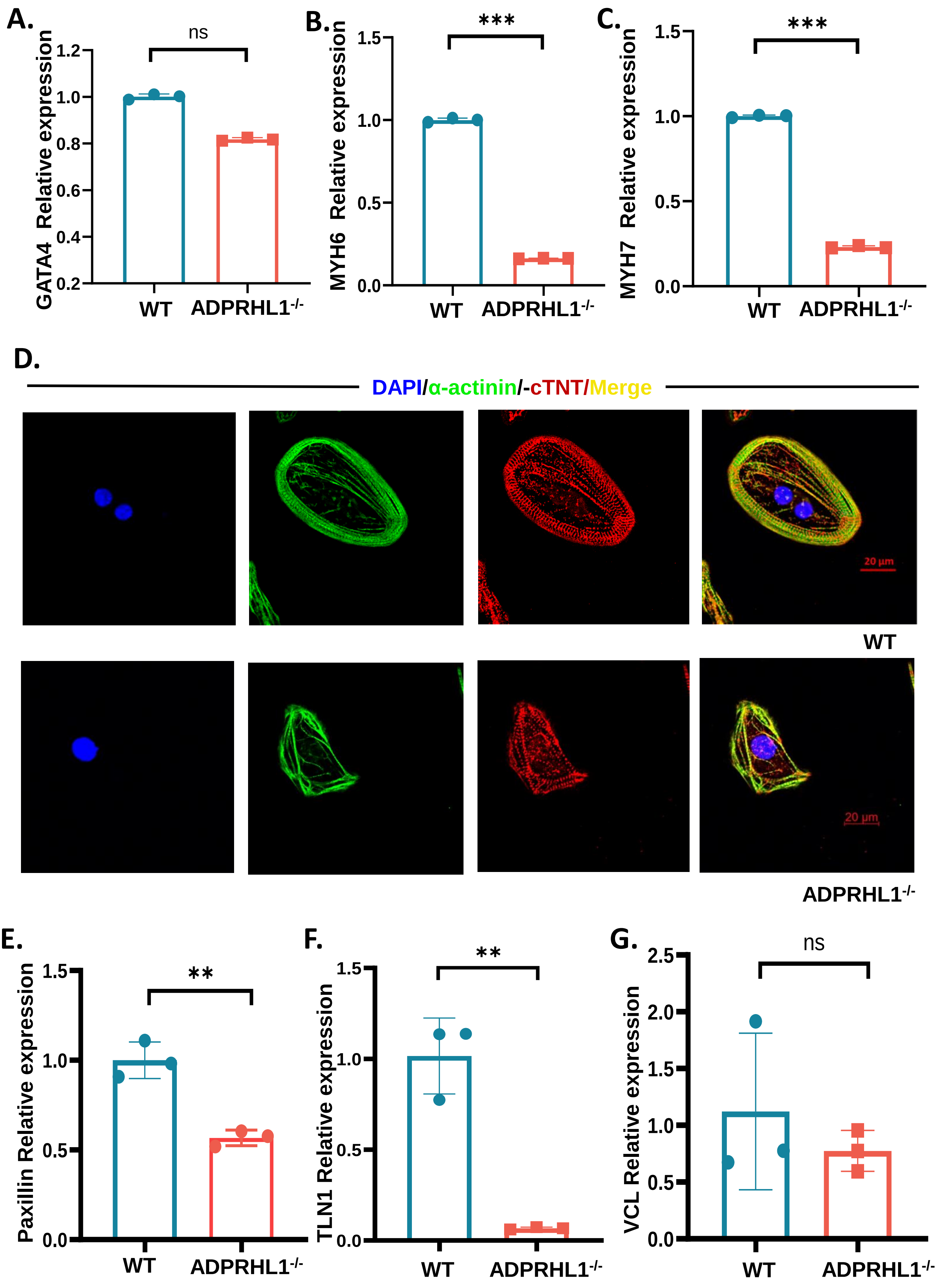

Supplementary figure 3

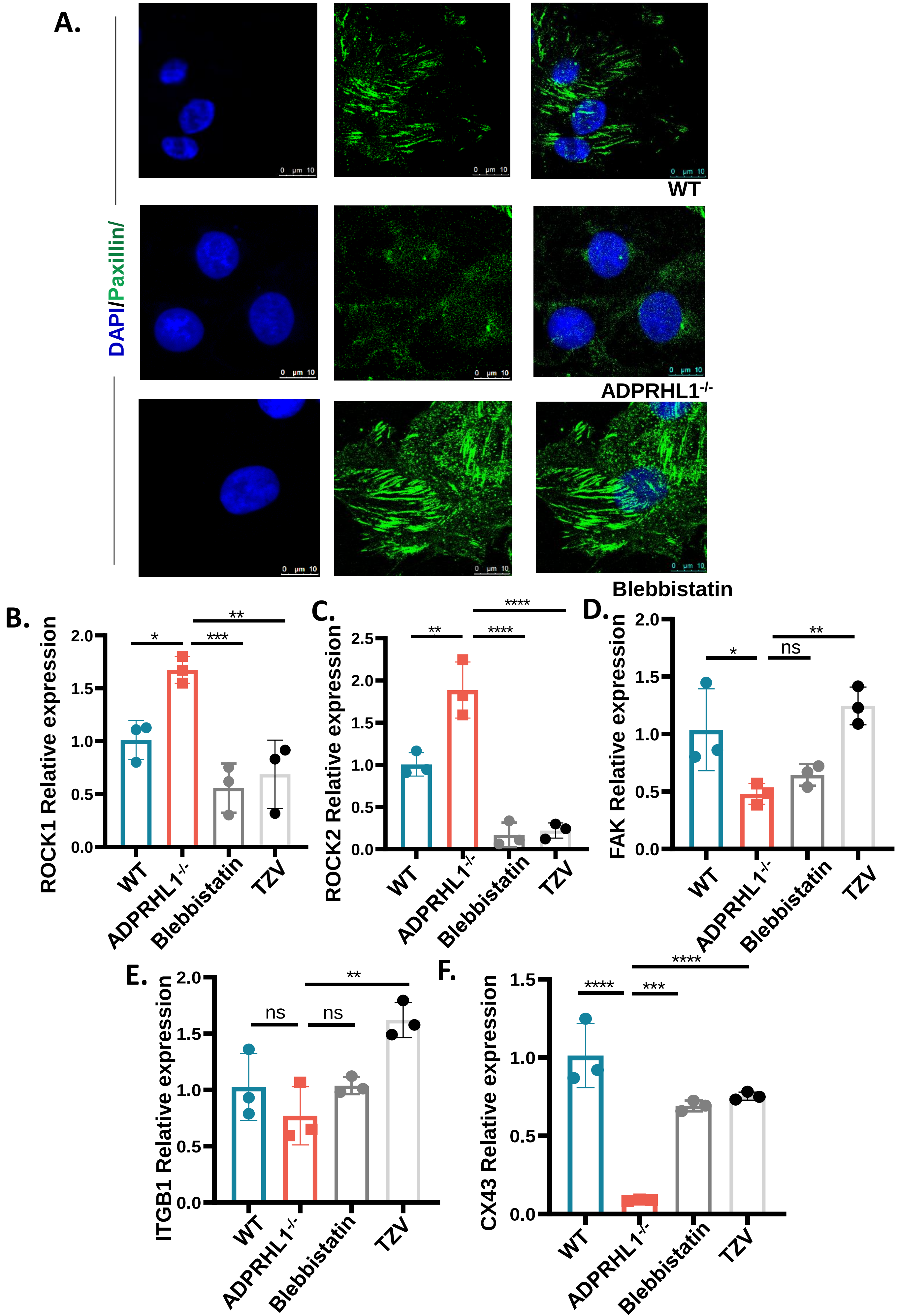

Supplementary figure 4

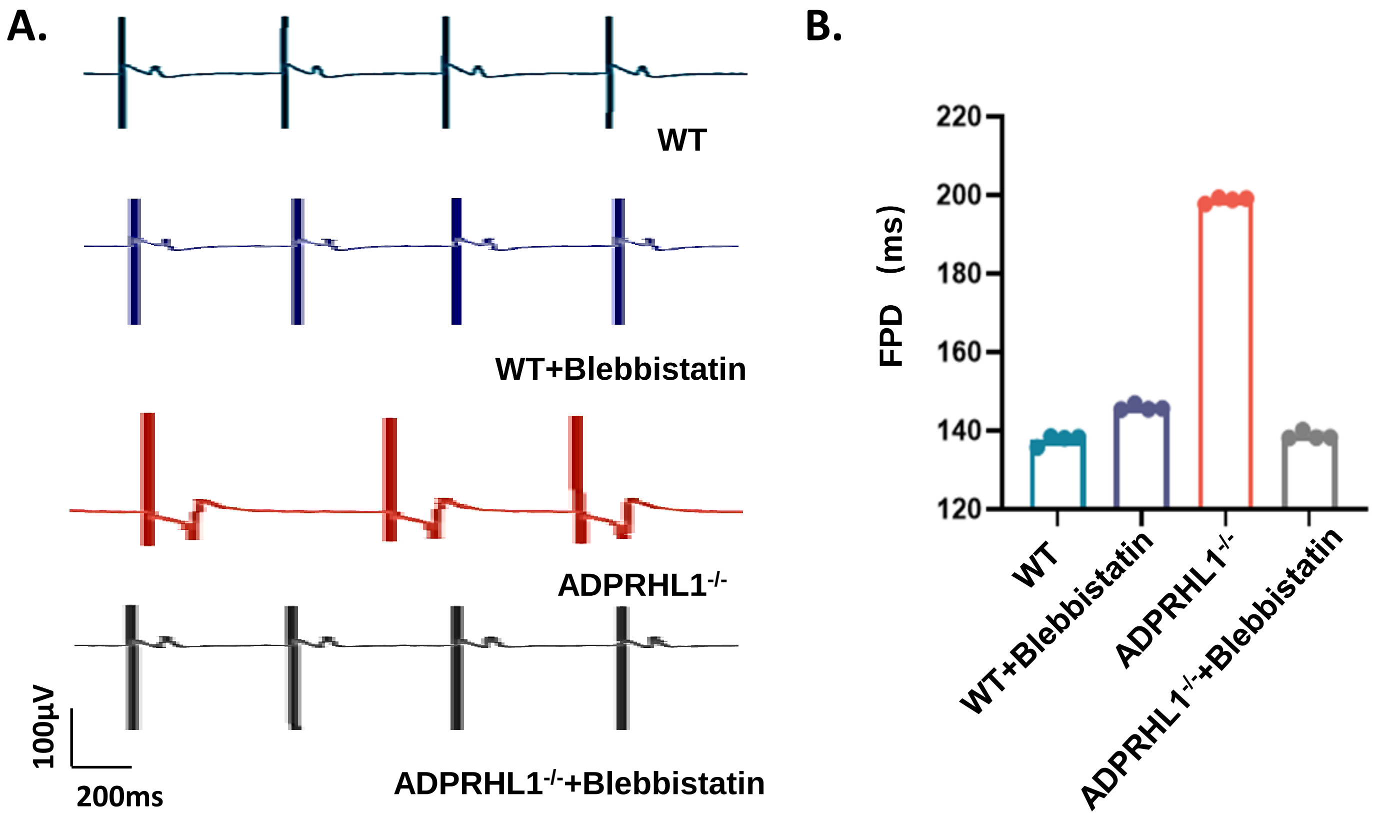

Supplement: Supplementary file 1 — Additional file 1. Supplementary figure 1. Identification of pluripotency markers and cardiomyocyte markers. (A) Schematic diagram of the CM differentiation method. (B) The WT and KO cells were tested for mycoplasma.(C, D and E) Quantification q-PCR analysis of SOX2, OCT4 and NANOG normalized by GAPDH in WT and KO at day 20. (F and G) Quantitative q-PCR analysis of Mesp1 and DKK1 normalized by GAPDH in WT and KO cells at day 2. (H, I and J) Quantitative q-PCR analysis of TBXT, NKX2.5 and TNNT2 normalized by GAPDH in WT and KO cells at day 4. Supplementary figure 2. Cardiomyocyte morphology and adhesion related indicators. (A, B and C) Transcriptional profiling data of GATA4, MYH6 and MYH7. (D) Immunofluorescence staining of WT-CMs and KO-CMs for α-actinin and cTNT. Scale bar, 20 μm. (E, F and G) Quantitative q-PCR analysis of Paxillin, TLN1 and VCL. Supplementary figure 3. Inhibition of the ROCK pathway rescues the cell adhesion phenotype of KO-CMs. (A) Immunofluorescence staining of WT-CMs, KO-CMs, and KO-CMs treated with Blebbistatin for paxillin. Scale bar, 10 μm (B) Quantitative q-PCR analysis of ROCK1, ROCK2, FAK, PXN, ITGB1 and Cx43 normalized by GAPDH in WT-CMs, KO-CMs and KO-CMs treated with Blebbistatin at day 20. Supplementary figure 4. Blebbistatin improves electrical conduction and calcium transients of KO-CMs. (A and B) MEA detection of the field potential duration of WT-CMs, KO-CMs, WT-CMs treated with Blebbistatin and KO-CMs treated with Blebbistatin at day 20, (n = 3 cells per group). [file 13287_2023_3507_MOESM1_ESM.pdf]
